# Computational Modeling of Cardiac Electrophysiology with Human Realistic Heart–Torso Model

**DOI:** 10.3390/bioengineering12040392

**Published:** 2025-04-06

**Authors:** Chen Yang, Yidi Cao, Peilun Li, Yanfei Yang, Min Xiang

**Affiliations:** 1 Key Laboratory of Ultra-Weak Magnetic Field Measurement Technology, Ministry of Education, School of Instrumentation and Optoelectronic Engineering, Beihang University, Beijing 100191, China; yc1995@buaa.edu.cn (C.Y.); caoyidi_buaa@buaa.edu.cn (Y.C.); yanfeiyang@buaa.edu.cn (Y.Y.); 2 Zhejiang Provincial Key Laboratory of Ultra-Weak Magnetic-Field Space and Applied Technology, Hangzhou Innovation Institute, Beihang University, Hangzhou 310051, China; 3 State Key Laboratory of Traditional Chinese Medicine Syndrome, National Institute of Extremely-Weak Magnetic Field Infrastructure, Hangzhou 310028, China; lpl194514@163.com; 4 Hefei National Laboratory, Hefei 230088, China

**Keywords:** bidomain model, body surface potential maps (BSPMs), electrocardiogram (ECG), Fitzhugh–Nagumo (FHN) model

## Abstract

The electrocardiogram (ECG) has long been considered the non-invasive gold standard in diagnosing heart diseases. However, its connection with the cardiac molecular biology remains somewhat unclear. Therefore, modeling the electrophysiological behavior of the heart provides an important theoretical complement to clinically observable data. This study employed an electrophysiological model, integrating a bidomain model with the Fitzhugh–Nagumo (FHN) model, to compute an ECG and body surface potential maps (BSPMs). Parameters from previous studies were simulated individually for the cardiac domain. A specific set of parameters was selected based on comparisons of the morphology of the 12-lead ECG. The effect of the heart position relative to the torso on the 12-lead ECG was analyzed using a simplified whole-heart model to approximate the realistic heart position within the torso. Significant waveform changes were observed in leads VIII and aVL, as compared to other leads. This study employed a realistic heart–torso model, in contrast to earlier studies. External stimuli were incorporated into the original electrophysiological model to account for the electrical isolation between the atria and ventricles. The morphology of the simulated 12-lead ECG closely matched that of clinically observed data.

## 1. Introduction

The human body is often viewed as a vast volume conductor, within which the heart is suspended. The electrical excitation of the heart originates from the sinoatrial node and then spreads through the atria. Atrial depolarization subsequently extends to the atrioventricular node before ultimately reaching the bundle branches or Purkinje fibers through the His bundle. During the cardiac electrical activity cycle, a portion of the heart tissue undergoes depolarization, whereas another portion remains at rest or polarized, resulting in charge separation or a dipole. The moving dipole induces an electric current flow in the body fluid surrounding both ends of the heart, leading to a fluctuating electric field throughout the body [1]. The electric field surrounding the heart can be detected by positioning electrodes on the surface of the body. The strength of the electric field is contingent upon the orientation of the electrodes relative to the ends of the dipole. The voltage that gives rise to these electric fields is the underlying reason for the generation of an electrocardiogram (ECG).

An ECG records the changes in cardiac electrical activity over time, which are measured by placing electrodes on the surface of the body. The ECG provides a cumulative signal of changes in the transmembrane potential caused by the depolarization and repolarization of cell populations during each cardiac cycle [1]. The 12-lead ECG is widely regarded as the non-invasive gold standard for the diagnosis of various heart diseases. However, despite being the most common tool in medicine today, the relationship between the ECG and cardiac molecular biology remains somewhat unclear. Therefore, realistic ECG simulations with mathematical models are crucial for research aiming to connect cellular and molecular physiology with clinically observable signals [2].

In the past few decades, the mathematical modeling of cardiac electrical activity has emerged as a prominent research method in the field of cardiac electrophysiology, with the advantage of providing a controlled environment, enabling the study of how changes in certain parameters quantitatively affect the entire system [3,4]. Meanwhile, the mathematical modeling of cardiac electrical activity is considered a crucial tool in understanding both cardiac function and diseases [5,6,7]. Cardiac electrical activity forms the foundation of cardiac function, leading researchers to conduct extensive studies on it at the cellular, tissue, and organ levels. In the multi-scale mathematical modeling of cardiac electrophysiology, the smallest physiological structure considered is typically based on ion channels in the cell membrane. Hodgkin and Huxley pioneered the use of mathematical equations to model ion currents and simulate the action potential of neurons [8]. Ten Tusscher and Panfilov developed a human ventricular cell action potential model that simulates intracellular calcium processing and heterogeneous action potential propagation, including endocardial, mid-myocardial, and epicardial [9,10]. Noble modeled Purkinje fibers by modifying the neural models originally developed by Hodgkin and Huxley [11]. When modeling myocardial cells as a functional syncytium, the stimulation and propagation of excitement can be viewed as a reaction–diffusion problem. In this case, the electrophysiological behavior of the heart can be modeled using bidomain and monodomain equations. The bidomain model is regarded as a continuous approximation of the myocardium, comprising two coexisting spaces, the intracellular domain and the extracellular domain, which are coupled together. When it is assumed that the intracellular and extracellular conductivities are proportional, the bidomain model can be simplified into a monodomain model [12,13].

This paper is organized as follows. Section 2 details the construction of geometric models, including a 3D simplified whole-heart model and a realistic heart–torso model. Section 3 introduces the model for cardiac electrophysiological simulation, based on a bidomain model combined with the FHN model, and clarifies the boundary conditions. Section 4 discusses the numerical results. Firstly, we selected parameters for the heart region model, and the simulated 12-lead ECG results exhibited a morphology and time course similar to those observed in a clinical ECG. Secondly, we conducted electrophysiological simulations using a 3D simplified whole-heart model to study the impact of heart position changes relative to the torso on the 12-lead ECG. Finally, we applied the electrophysiological model to a realistic heart–torso model and introduced external stimuli to address the electrical isolation between the atria and ventricles.

## 2. Geometric Model Generation

**Figure 1:**
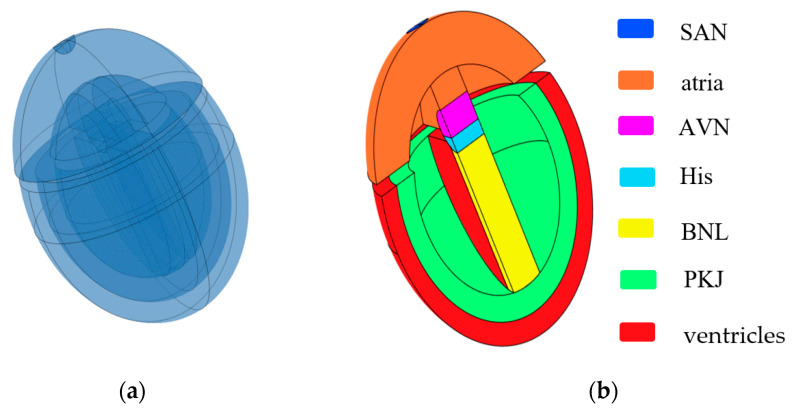
Simplified 3D whole-heart model. (**a**) Semitransparent view of the heart model; (**b**) cross-sectional view of the heart model. Different colored bars represent different subdomains of the heart.

Inspired by the work of Sovilj et al. [14,15] and Biasi et al. [16], we constructed a 3D simplified whole-heart model using COMSOL Multiphysics 6.2. This model is designed to adapt to changes at the cellular level and generate ECG waveforms. The 3D simplified whole-heart model, as shown in Figure 1, comprises the atrial and ventricular myocardium, along with the principal components of the cardiac conduction system. These components include the sinoatrial node (SAN), slow-conducting atrioventricular node (AVN), His bundle (His), bundle branches (BNL), and Purkinje network (PKJ). The overall structure comprises three nested ellipsoidal shells, separated into upper and lower parts by two interconnected cylinders. The upper part represents the atria, while the lower part contains two nested ellipsoidal shells that symbolize the ventricular muscle and Purkinje fibers. The outer ellipsoidal shell corresponds to the ventricular muscle, and the inner shell represents the Purkinje fibers. The two cylinders, respectively, represent the AVN and the His bundle. A sphere embedded on the right side of the atria represents the SAN, and a distinct cuboid models the bundle branches.

The dimensions of all subdomains closely approximate the real human anatomical structure from the Visible Human Project gallery. Different subdomains exhibit distinct myocardial cell characteristics and conductivity. There exists an electrical isolation gap between the atria and ventricles. However, the connection between the AVN and the His bundle is excluded at the septum connection.

The numerical simulation of the simplified 3D heart–torso model in this study was performed using COMSOL Multiphysics 6.2. The mesh consists of 68,555 tetrahedral elements, with 140,227 degrees of freedom solved at each time step. To improve the computational accuracy, the “pre-defined selection finer” mesh option was applied to the heart domain, while the “pre-defined selection refine” option was used for the torso domain, dividing the mesh into smaller units to more accurately capture changes in the electric field. The time step for the transient simulation was 0.001 s, with a time range from 0 to 2 s. The solver utilizes a fully coupled direct solver.

**Figure 2:**
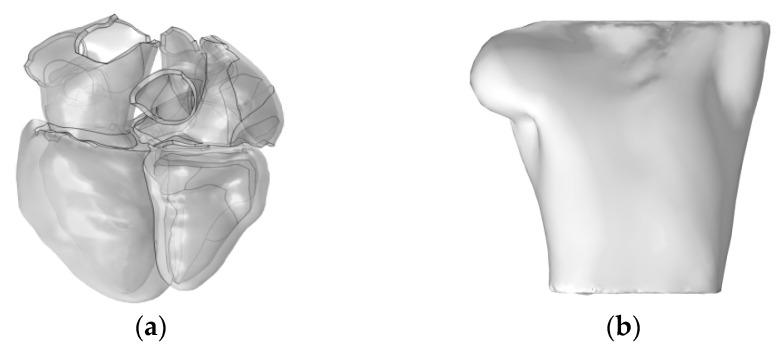
(**a**) Realistic whole-heart model; (**b**) 3D torso model.

Subsequently, we utilized the high-resolution heart model developed by Hoogendoorn et al., which is based on multi-slice CT (MSCT) imaging data [17]. This model constructs detailed cardiac anatomical structures, including the atria, ventricles, and large blood vessels, through semi-automatic segmentation and 3D reconstruction techniques. In the heart model, the left and right atria and the ventricles are discretely separated and unconnected, forming three distinct solution domains, as shown in Figure 2a. The torso domain incorporates a realistic RIUNE torso geometry (RIUNET, http://hdl.handle.net/10251/55150, accessed on 27 May 2024) [18], which was obtained from the publicly available online repository of the Centre for Integrative Biomedical Computing (CBIC) from the University of Utah [19]. The model is assumed to be isotropic, with a torso conductivity of 0.2 S/m [14,16,20], as illustrated in Figure 2b.

The mesh for the realistic 3D heart–torso numerical simulation consists of 44,790 tetrahedral elements, with 109,540 degrees of freedom solved at each time step. The heart domain employs the “pre-defined selection fine” mesh option, while the torso domain utilizes the “pre-defined selection normal” mesh option. The time step for the transient simulation is 0.007 s, with a time range from 0 to 0.6 s. The solver utilizes a fully coupled direct solver.

## 3. Mathematical Modeling

### 3.1. Bidomain Model Incorporated with FHN Model

The mathematical model employed in this study is based on the bidomain reaction–diffusion model initially proposed by Tung [21]. In electrophysiology, the bidomain model simulates the propagation of electrical activity within myocardial tissue and the response to external stimuli in cardiac tissue. In the model, the cardiac domain is divided into two overlapping, independent, homogeneous domains: the intracellular space and the extracellular space. The intracellular and extracellular potentials at each point within the computational domain are governed by the bidomain equation. This equation is derived by interpreting the intracellular and extracellular conductivities as an “average” conductivity and applying Ohm’s law and the law of charge conservation to these domains [22,23]. Let the current densities inside and outside the cell be represented as $J_{i}$ and $J_{e}$, respectively. According to Ohm’s law, the intracellular and extracellular potentials ($V_{i}$ and $V_{e}$) are related to the current densities by [24]

(1) $$\left\{\begin{array}{l} J_{i}=\sigma i\nabla V_{i}\\ J_{e}=\sigma_{e}\nabla V_{e} \end{array}\right.$$

where $\sigma_{i}$ and $\sigma_{e}$ represent the intracellular and extracellular conductivities, respectively.

The transmembrane current $I_{m}$, which flows through the cell membrane, can be derived from the principle of current conservation [25]:

(2) $$\left\{\begin{array}{l} I_{m}=\nabla \cdot J_{i}\\ I_{m}=-\nabla \cdot J_{e} \end{array}\right.$$

The transmembrane potential $V_{m}$ is defined as the difference between the intracellular and extracellular potentials:

(3) $$V_{m}=V_{i}-V_{e}$$

The cell membrane can be modeled as a parallel circuit comprising a resistor and a capacitor. The transmembrane current can thus be expressed as the sum of the capacitive current and the ionic current $I_{ion}$ [26]:

(4) $$\beta C_{m}\frac{\partial V_{m}}{\partial t}+\beta I_{ion}=I_{m}$$

where $C_{m}$ represents the membrane capacitance per unit area of the cell membrane, and β denotes the ratio of the cell membrane surface area to its volume.

By combining the above equations and assuming isotropy conductivity for both the intracellular and extracellular spaces, the following equation can be obtained:

(5) $$\left\{\begin{array}{l} \frac{\partial V_{e}}{\partial t}-\frac{\partial V_{i}}{\partial t}+\nabla \cdot -\sigma_{e}\nabla V_{e}=I_{ion}\\ \frac{\partial V_{i}}{\partial t}-\frac{\partial V_{e}}{\partial t}+\nabla \cdot -\sigma_{i}\nabla V_{i}=-I_{ion} \end{array}\right.$$

The Fitzhugh–Nagumo (FHN) model is a commonly used implementation of reaction–diffusion systems for the simulation of the propagation of electrical impulses in cardiac tissue [27]. When solving the original FHN model, the waveform of the solution will exhibit the phenomenon of hyperpolarization, which is not a characteristic of cardiac action potential and may have a negative impact on the recovery characteristics of the model, especially in complex reentry activation patterns. Therefore, Rogers and McCulloch modified the model accordingly [28].

By integrating the bidomain model with the FHN model, for the SAN, the expression is as follows [27]:

(6) $$\left\{\begin{array}{l} I_{ion}=kc_{1}(V_{m}-B)(a-\frac{V_{m}-B}{A})(1-\frac{V_{m}-B}{A})+kc_{2}v\\ \frac{\partial v}{\partial t}=ke(\frac{V_{m}-B}{A}-dv-b) \end{array}\right.$$

In other subdomains of the heart, the expression is formulated as [28]

(7) $$\left\{\begin{array}{l} I_{ion}=kc_{1}(V_{m}-B)(a-\frac{V_{m}-B}{A})(1-\frac{V_{m}-B}{A})+kc_{2}v(V_{m}-B)\\ \frac{\partial v}{\partial t}=ke(\frac{V_{m}-B}{A}-dv-b) \end{array}\right.$$

where $V_{m}$ represents the transmembrane potential; v represents a recovery variable, which controls the cell refractory period. k, $c_{1}$, $c_{2}$, A, B, and e are parameters that regulate the shape of the cell action potential curve, respectively. k represents the time scale of the model; $c_{1}$ and $c_{2}$ control the amplitude of ion current excitation and the refractory period. B represents the membrane potential; A controls the amplitude of the action potential; e controls the duration of the action potential; and a, b, and d are intermediate parameters.

### 3.2. Simulation of Standard Twelve-Lead ECG

**Figure 3:**
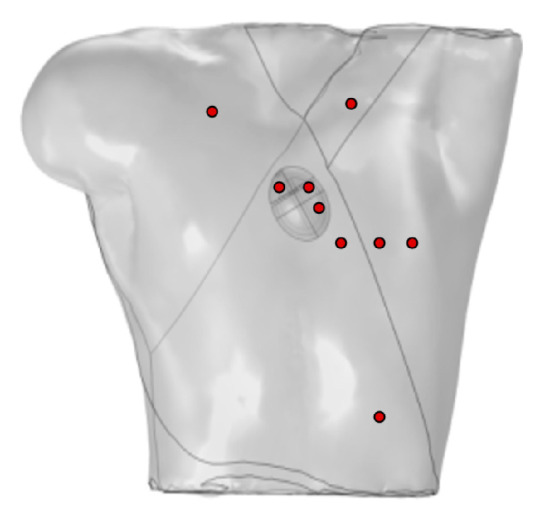
Probe placement for calculation of 12-lead ECG, where red circles represent probes V_GND_ represents the reference electrode, so the potential at this point is set to 0 V.

Ten probes were positioned on the surface of the torso to simulate the generation of a standard 12-lead ECG, as shown in Figure 3. Four probes were placed at the vertices of the torso, with the probe at the lower-right vertex set to 0 potential, satisfying the Dirichlet boundary condition, and the remaining probes placed on the anterior chest.

Let us consider the torso of the body as an equilateral triangle, thus forming the famous Einthoven triangle. Electrodes are placed at each vertex of the triangle, which can be located on the wrists and left ankle or on the shoulder and the lower part of the torso. This configuration allows for the calculation of the Einthoven leads and Goldberger-augmented leads.

(8) $$\begin{array}{l} VI=V_{L}-V_{R}\\ VII=V_{F}-V_{R}\\ VIII=V_{F}-V_{L}\\ aVR=\frac{\left(2V_{R}-V_{L}-V_{F}\right)}{2}\\ aVL=\frac{\left(2V_{L}-V_{R}-V_{F}\right)}{2}\\ aVF=\frac{\left(2V_{F}-V_{R}-V_{L}\right)}{2} \end{array}$$

The other six probes are used to directly calculate the precordial leads, computed as the difference between each precordial electrode and Wilson’s central terminal, defined as

(9) $$V_{CT}=\frac{V_{L}+V_{R}+V_{F}}{3}$$

To simulate the cardiac electrical activity, it is necessary to choose model parameters corresponding to specific regions of the heart. For this purpose, three models were compared to select a set of parameters that best matched the ECG characteristics. The parameter values are shown in Table 1.

For the initial values of the model variables, at the SAN, the intracellular potential is set to −0.06 V, and, at the other subdomains of the heart, the intracellular potential is set to −0.085 V. Additionally, all extracellular potentials and recovery variables are set to 0.

The heart and torso domains are discretized using second-order tetrahedral elements, with quadratic Lagrange element functions that are well suited for complex geometries and offer higher computational accuracy. The TMP distribution is determined using the Galerkin method and applied to the cardiac electric field model to obtain the BSPM distribution.

### 3.3. Model Boundary Conditions

The passive volume conductor in this study, i.e., the torso, follows the Laplace equation:

(10) $$\nabla \cdot \left(-\sigma_{b}\nabla V\right)=0$$

where $\sigma_{b}$ is the torso conductivity, and V represents the torso potential.

When applying flux-free boundary conditions to the intracellular potential to enclose and approximate the intracellular space, Neumann boundary conditions are imposed [2].

(11) $$n\cdot \sigma_{i}\nabla V_{i}=0$$

All external boundaries of the torso are considered as electrical insulation, where the vertical component of the current density is zero and can be expressed as

(12) n⋅J=0

The continuity boundary conditions require the internal boundary of the torso in contact with the heart to be set as follows:

(13) $$V=V_{e}$$

where $V_{e}$ represents the extracellular potential of the myocardium.

### 3.4. Mesh Convergence Analysis

We first identified key indicators for the evaluation of mesh convergence: the transmembrane potentials at specific points on the left ventricular epicardium and endocardium for the cardiac domain, and the P-wave, QRS-wave, and T-wave amplitudes of the 12-lead ECG for the torso domain. The initial mesh for the heart domain is set to “fine” and for the torso domain to “normal”. Mesh refinement proceeds by increasing the heart domain mesh from “fine” to “finer” and the torso domain mesh from “normal” to “fine”. The relative error of the key indicators is calculated between adjacent mesh settings. If the error is less than the convergence threshold (e.g., 1%), the refinement stops. The test results are shown in Table 2.

Based on the test results in Table 2, the relative error in Test 2 is less than 1%, indicating that the results from Test 1 are close to convergence, and the further refinement of the heart domain (from “fine” to “finer”) has little effect on the results. In contrast, the relative error in Test 3 is greater than 1%, suggesting that refining the torso domain (from “normal” to “fine”) significantly impacts the results and that the torso domain’s mesh resolution has not yet converged. However, the results from Test 1 already meet the accuracy requirements and offer low computational costs. Further refinement in Test 3 did not improve the results but increased the computational costs. To balance efficiency and accuracy, we selected the mesh resolution from Test 1. An additional Test 4, where the torso domain was further refined, still resulted in a significant error (>1%).

## 4. Results

### 4.1. Selection of Model Parameters

Different cardiac models yield varying ECG simulation results due to differences in their regional parameters. To achieve simulations that closely resemble a real ECG in morphology, selecting specific parameter models is essential. In this study, three parameter models are selected to calculate the 12-lead ECG separately, which are labeled Model 1, Model 2, and Model 3, respectively.

**Figure 4:**
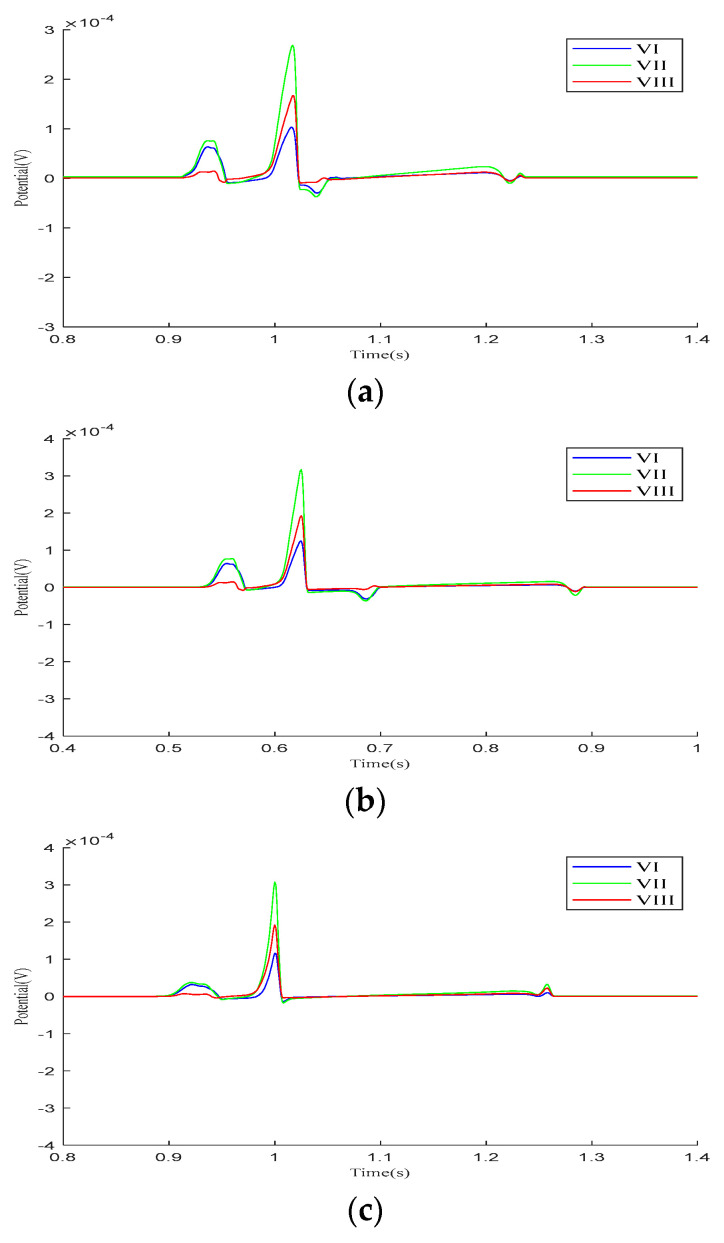
Einthoven lead (VI, VII, and VIII) results for different parameter models. (**a**) Model 1, (**b**) Model 2, (**c**) Model 3.

We analyzed the Einthoven leads (VI, VII, VIII), Goldberger-augmented leads (aVR, aVL, aVF), and precordial leads (V1–V6) across the three parameter models. Figure 4 presents the calculated results of the Einthoven leads during a cardiac electrical conduction cycle. In Model 1, leads VI and VII display broader S-waves, lead VIII shows normal P- and QRS-waves, and all three leads exhibit inverted T-waves. In Model 2, inverted T-waves are observed across all three leads. Following ventricular depolarization and at the onset of repolarization, a negative deviation occurs between the QRS- and T-waves, with an amplitude exceeding the T-wave peak. In Model 3, both the QRS-waves and T-waves exhibit overall normal progression.

**Figure 5:**
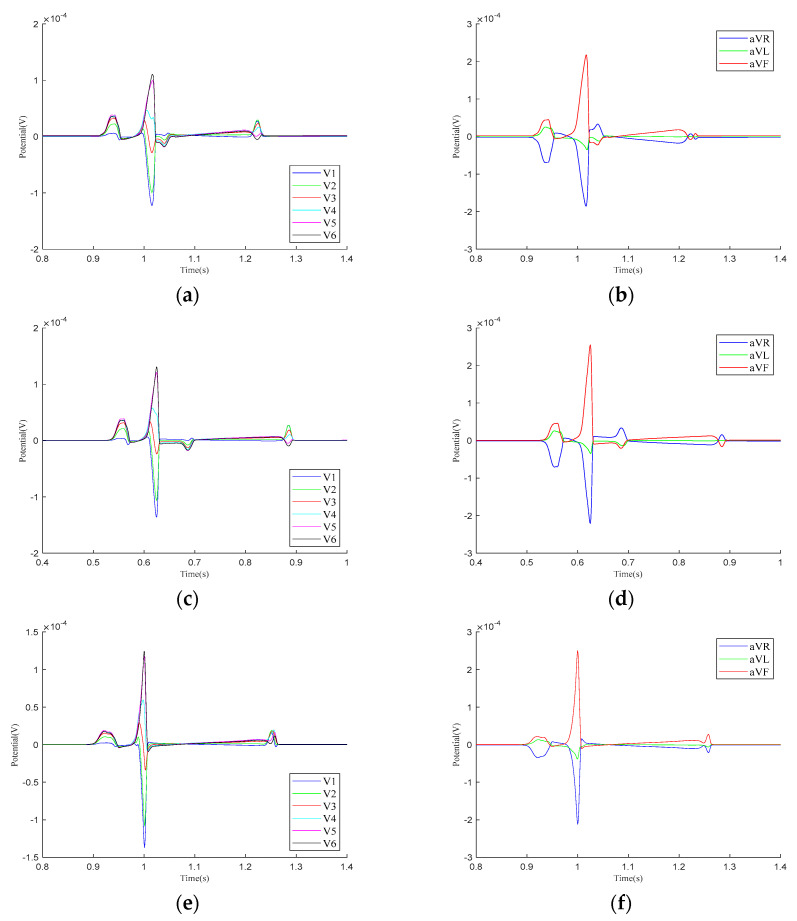
Calculation results for precordial leads and Goldberger-augmented leads. (**a**) V1–V6 leads of Model 1. (**b**) aVR, aVL, and aVF leads of Model 1. (**c**) V1–V6 leads of Model 2. (**d**) aVR, aVL, and aVF leads of Model 2. (**e**) V1–V6 leads of Model 3. (**f**) aVR, aVL, and aVF leads of Model 3.

Figure 5 presents the calculation results for the precordial leads and Goldberger-augmented leads across the three parameter models. In Model 1, T-wave inversion is observed in leads V5 and V6, while, in lead aVR, the T-wave is positive, opposing the directions of the P-wave and R-wave. In lead aVF, the T-wave shows a negative deviation along the vertical axis and opposes the P-wave and R-wave. In Model 2, negative waveform deviations between the QRS- and T-waves are noted in leads V1–V6, with the T−wave inversion persisting in leads V5 and V6. The behaviors in leads aVR and aVF are consistent with those in Model 1. In Model 3, the simulation results for both the V1–V6 leads and the aVR, aVL, and aVF leads align with realistic ECG morphologies. Based on these findings, the regional parameters of Model 3 were selected.

### 4.2. Impact of Heart–Torso Relative Position on Twelve-Lead ECG

**Figure 6:**
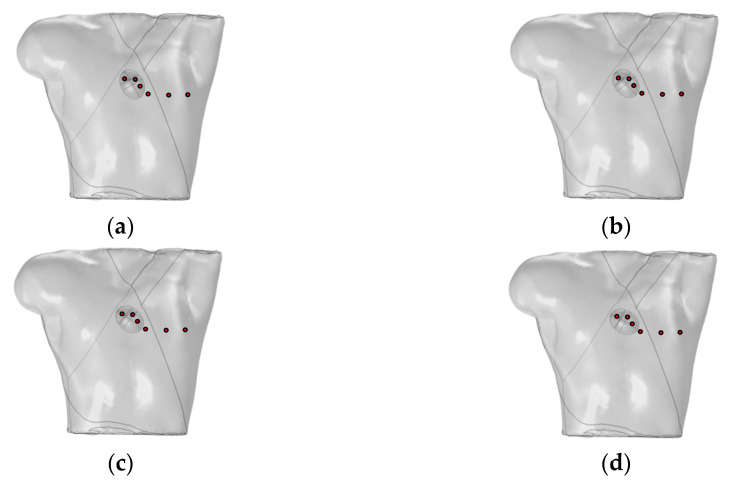
(**a**) Initial position of the heart relative to the torso model; (**b**) heart rotates counterclockwise by 10° relative to the torso model; (**c**) heart rotates counterclockwise by 20° relative to the torso model; (**d**) heart rotates counterclockwise by 30° relative to the torso model. where red circles represent probes.

Mincholé et al. obtained the anatomical structures of the ventricles and torso from clinical standard magnetic resonance imaging (MRI). Utilizing these data, they developed an electrophysiological model to simulate the 12-lead ECG and quantify the QRS-wave morphology. Their research demonstrated that the heart’s position within the torso significantly influences the QRS-wave morphology in the precordial leads [29]. This study employed an existing electrophysiological model integrating the bidomain model with the FHN model to analyze how changes in the heart position relative to the torso influence the 12-lead ECG. As shown in Figure 6, the heart rotates counterclockwise by 10°, 20°, and 30° relative to the torso from its initial position. The 12-lead ECG signal at the heart’s initial position was used as a reference signal.

**Figure 7:**
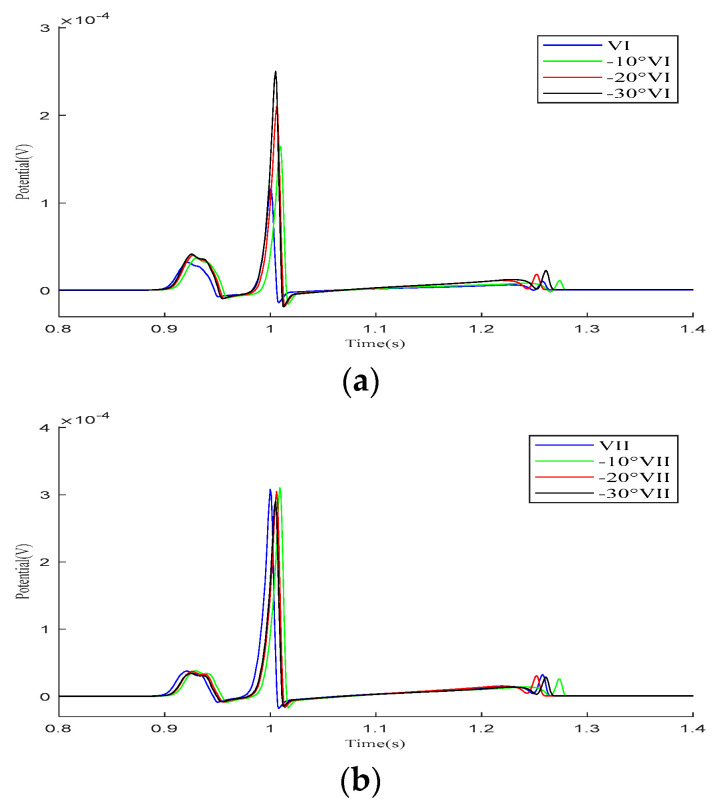

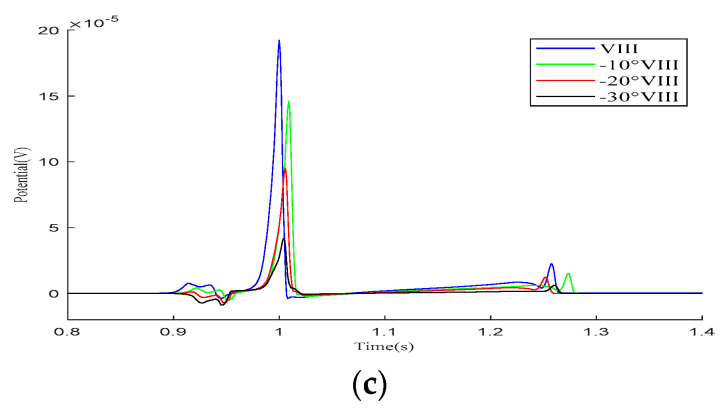
Simulation results for the Einthoven leads during heart rotation relative to the torso model. (**a**) Simulation results for lead VI; (**b**) simulation results for lead VII; (**c**) simulation results for lead VIII. Here, −10°, −20°, and −30° refer to counterclockwise rotation of 10 degrees, 20 degrees, and 30 degrees, respectively.

As demonstrated in the simulation results for the Einthoven leads in Figure 7, when the heart rotates counterclockwise relative to the torso model, the overall morphology of leads VI and VII remains unchanged during the propagation of cardiac electrical activity. However, lead VIII shows a significant change, with the P-wave deflection shifting from the positive direction of the potential axis to the negative direction. The amplitude of the R-wave in lead VI gradually increases, whereas, in lead VIII, it gradually decreases.

**Figure 8:**
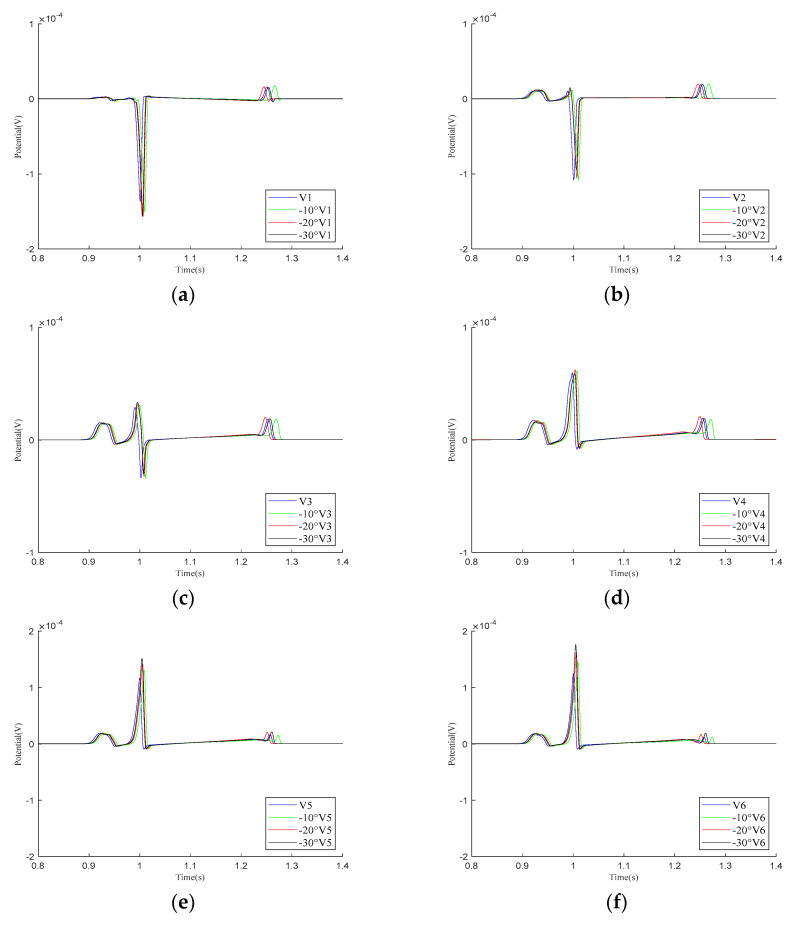
Simulation results for V1–V6 precordial leads during the rotation of the heart relative to the torso model are presented in (**a**–**f**), respectively. Here, −10°, −20°, and −30° refer to counterclockwise rotation of 10 degrees, 20 degrees, and 30 degrees, respectively.

As shown in the simulation results for the V1–V6 leads in Figure 8, within one cardiac pulse propagation cycle, the overall waveform changes of all leads are not significant. When the cardiac rotation angle changes, the R-wave amplitudes of leads V1, V5, and V6 slightly increase compared to the other leads.

**Figure 9:**
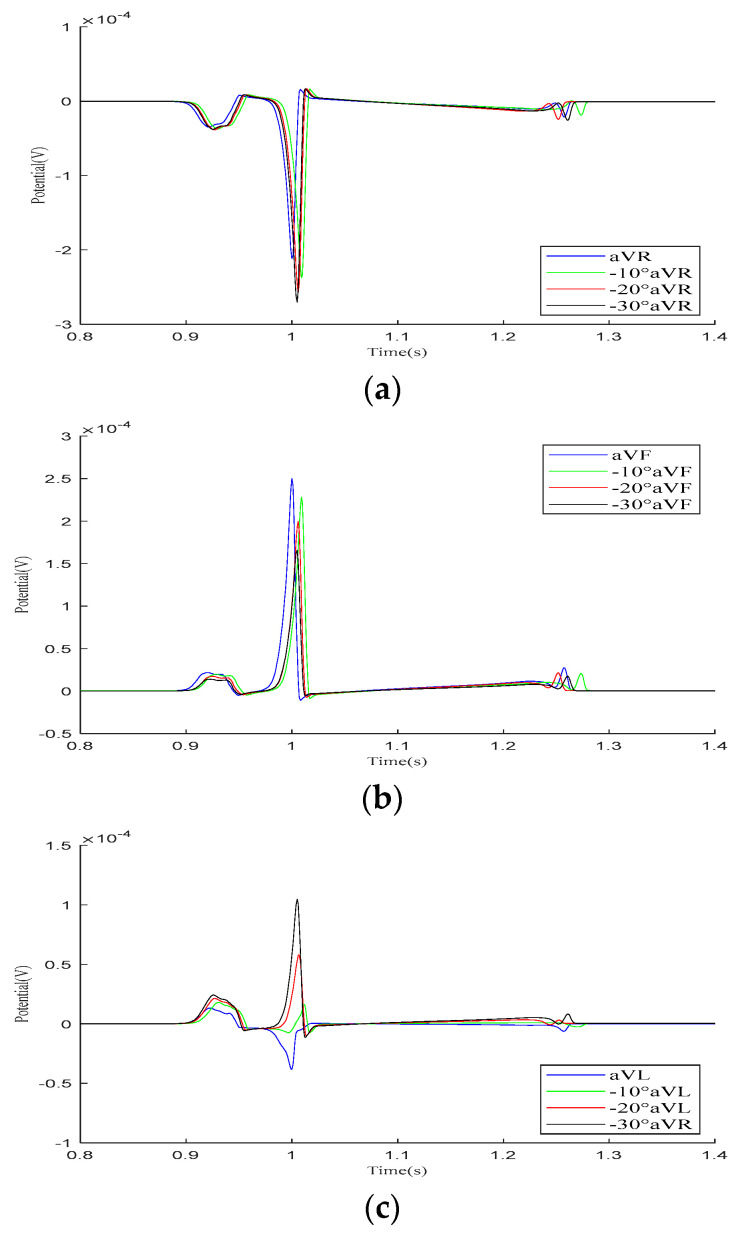
Simulation results for Goldberger-augmented leads during the rotation of the heart relative to the torso model are presented in (**a**–**c**), respectively. Here, −10°, −20°, and −30° refer to counterclockwise rotation of 10 degrees, 20 degrees, and 30 degrees, respectively.

In the Goldberger-augmented leads shown in Figure 9, the overall waveform morphology of the aVR and aVF leads does not change significantly. When the heart rotates counterclockwise relative to the torso, the R-wave amplitude in the aVR lead increases, while the R-wave amplitude in the aVF lead decreases. However, during this process, the waveform of the aVL lead changes significantly, with the R-wave direction shifting from an initial negative deviation along the potential coordinate axis to a positive deviation, with its amplitude gradually increasing.

Our simulation results demonstrate that variations in the relative position of the heart and torso models can alter the 12-lead ECG waveform. The most significant changes occur in the P-wave of lead VIII and the R-wave of lead aVL, although these variations remain within the normal range. Other leads mainly exhibit changes in the R-wave amplitude. In conclusion, when defining the relative position of the heart and torso, it is essential to orient the apex of the heart towards the left, lower, and anterior regions of the torso.

### 4.3. Electrophysiological Simulation Based on Realistic Heart–Torso Model

**Figure 10:**
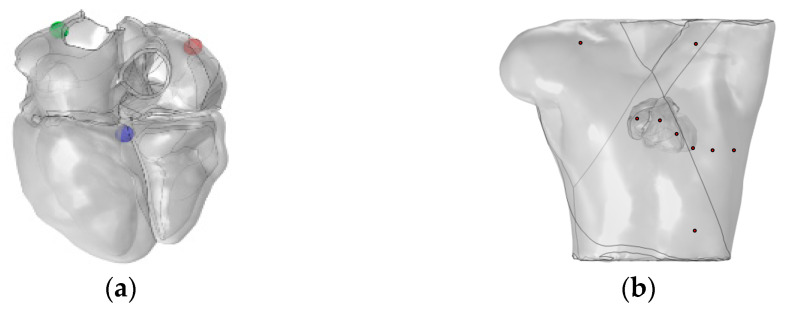
(**a**) Realistic heart model and locations for stimulation application. (**b**) Relative position of the heart–torso and probe placement. Red, green, and blue balls represent the SAN, BB, and AVN, respectively.

To validate the feasibility of integrating the bidomain reaction–diffusion model into the FHN model to compute the electrophysiological output of a realistic heart–torso model, a realistic heart model was constructed. Referring to [30], as depicted in Figure 10a, in order to sequentially stimulate the propagation of electrical signals in the heart, the atrial and ventricle pathways were modeled as three spherical delayed stimuli along the geometric shape of the heart that mimicked the inter-cardiac connections. For the right atrium, a single stimulus is applied at the SAN. In the left atrium, stimulation is applied to Bachmann’s bundle (BB). The stimulation starts at the SAN at t = 2 ms (red ball in Figure 10a). As the transmembrane potential wave front reaches BB (green ball in Figure 10a) at t = 28 ms, the stimulation current is triggered sequentially. This facilitates the propagation of electrical signals from the right atrium to the left atrium. At t = 150 ms, a stimulus is applied to the AVN (blue ball in Figure 10a). The stimulation current is 100 A, with a stimulation duration of 2 ms. The sampling duration is 600 ms, with a sampling interval of 1 ms. Figure 10b illustrates the position of the heart within the torso and the probe placement used to calculate the 12-lead ECG.

**Figure 11:**
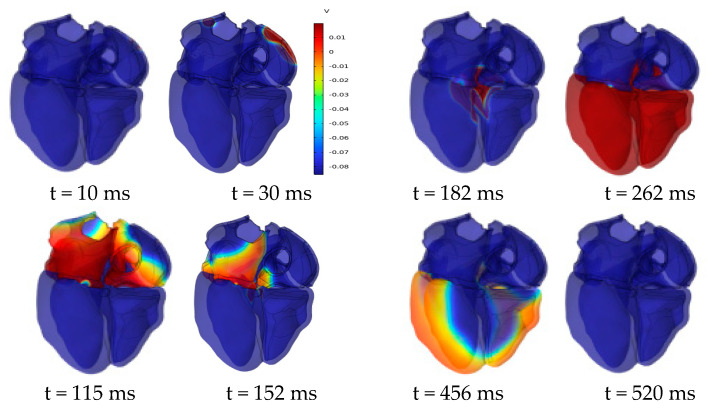
Dynamics of transmembrane potential throughout the cardiac cycle.

Figure 11 shows the evolution of the cardiac transmembrane potential (TMP) throughout the cardiac cycle, aligning with the expected results. The atria experiences depolarization and repolarization, with electrical signals originating from the SAN in the right atrium and propagating to the left atrium. Complete atrial depolarization occurs at around 115 ms after initial activation, and repolarization takes place at approximately 180 ms. Complete ventricular depolarization occurs at around 260 ms after initial activation, followed by complete repolarization at approximately 520 ms. The simulation results fall within the time course range of the TMP at a normal heart rate [31].

**Figure 12:**
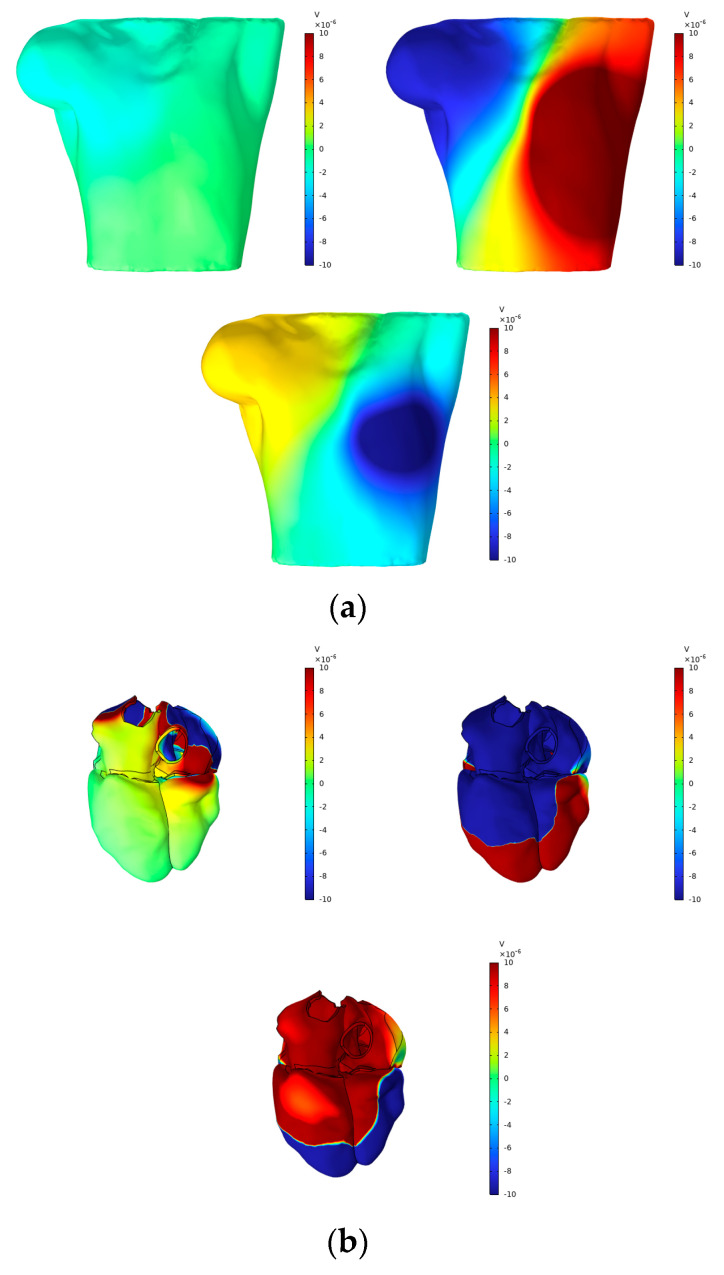
(**a**) BSPMs and (**b**) extracellular potentials associated with the P-wave, R-wave, and T-wave peaks.

**Figure 13:**
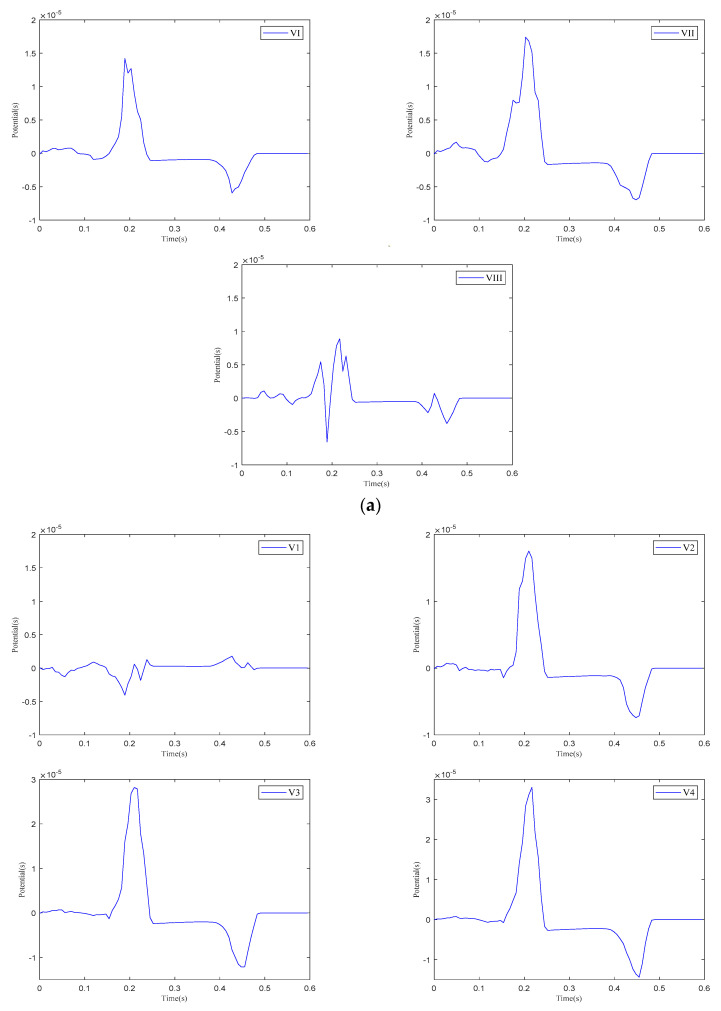

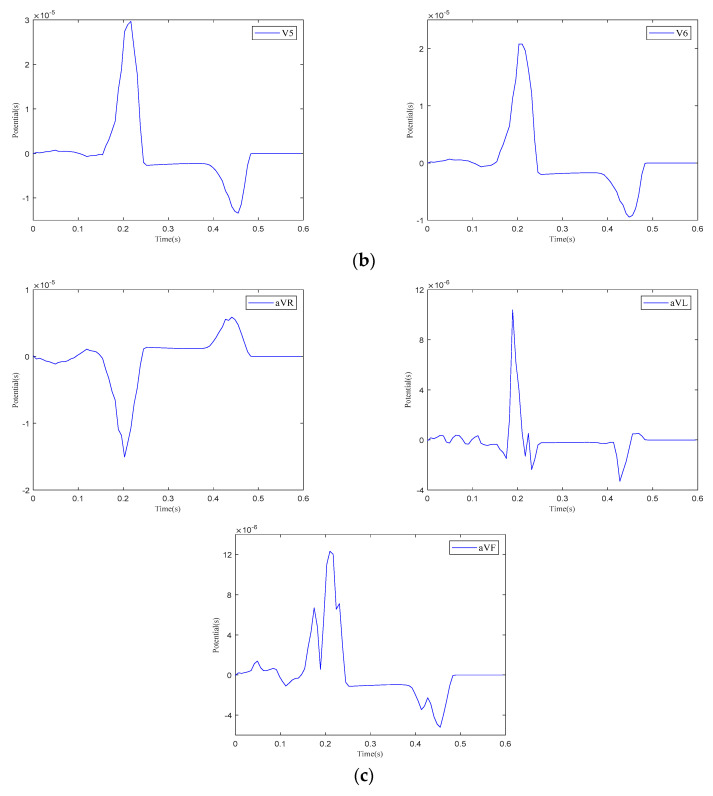
The 12-lead ECG. (**a**) Einthoven leads. (**b**) Precordial leads. (**c**) Goldberger-augmented leads.

According to Vieau and Iaizzo’s detailed explanation of cardiac axis vector changes during myocardial depolarization and repolarization [1], atrial depolarization under normal conditions generates axis vectors pointing from the upper right to the lower left. At the P-wave peak, the maximum torso surface potential occurs in the lower left corner, while the minimum value is observed in the upper right corner. The axial vector changes during ventricular depolarization and repolarization remain consistent, aligning the T-wave direction with the main direction of the QRS complex on the ECG. At the peaks of the R-wave and T-wave, the maximum torso surface potential is found in the lower left corner, while the minimum is located in the upper right corner. In this study, simple electrical stimulation of the ventricle induced repolarization, driving the endocardium to expand towards the epicardium. Consequently, the ECG exhibited T- and R-waves in opposite directions, with the maximum torso surface potential in the upper right and the minimum in the lower left. This phenomenon is illustrated in Figure 12 and Figure 13.

Figure 12 presents the BSPMs and extracellular potentials at the peaks of the P-wave, R-wave, and T-wave during the propagation of cardiac electrical activation waves. The changes in the epicardial potentials and the dynamic variations in the torso surface potentials remain well aligned throughout the cardiac electrical activity propagation process.

As shown in Figure 13, when current stimulation is applied to the SAN, BB, and AVN, a significant voltage pulse signal appears in lead 12 at the corresponding time points. The waveform morphology and time course of the ECG align with previously published research findings [31,32]. In leads VI, VII, aVF, and V4 to V6, the P-wave deflection is upward; in lead aVR, it is downward. The remaining leads exhibit bidirectional, inverted, or low-level signals. The main QRS-wave direction is upward in leads VI, VII, VIII, aVF, and V4 to V6, while it is downward in lead aVR.

**Figure 14:**
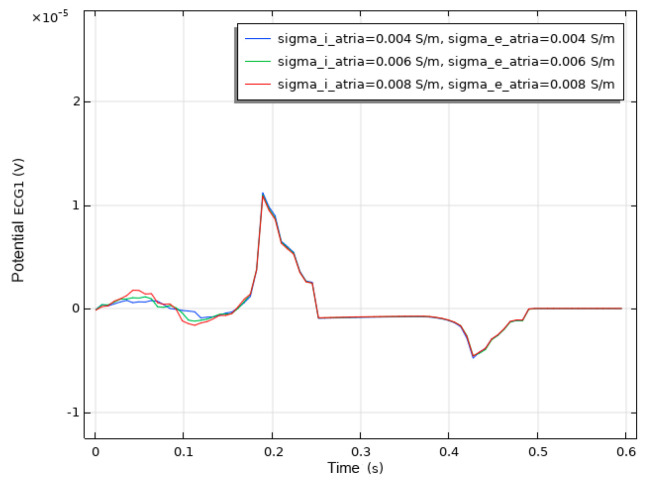
ECG waveform resulting from changes in the conductivity of atrial muscle cells.

**Figure 15:**
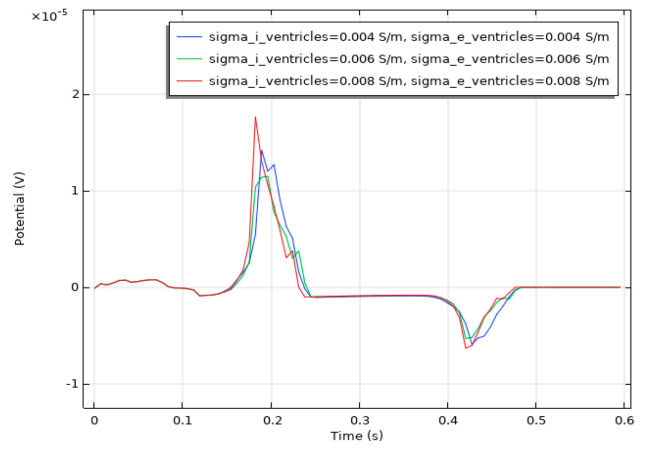
ECG waveform resulting from changes in the conductivity of ventricular muscle cells.

We conducted a sensitivity analysis to evaluate the impact of the intracellular and extracellular conductivity of myocardial cells on the simulation results. Firstly, we modified the intracellular and extracellular tissue conductivity of atrial myocytes within physiologically plausible ranges (e.g., based on references [14,15,16]) and examined the resulting changes in the ECG waveforms and activation patterns. As illustrated in Figure 14, an increase in intracellular and extracellular conductivity leads to a shorter P-wave duration and a higher amplitude. Subsequently, we modified the intracellular and extracellular tissue conductivity of ventricular myocytes and examined the resulting changes in the ECG waveforms and activation patterns. As shown in Figure 15, an increase in intracellular and extracellular conductivity results in a shorter duration for the QRS- and T-waves, along with a higher amplitude. However, the shape and duration of the ECG waveforms in Figure 14 and Figure 15 fall within a reasonable range. This analysis confirmed that our parameter choices were robust and physiologically reasonable.

## 5. Discussion

Modeling techniques based on the patient-specific cardiac anatomy and electrophysiology show great promise for clinical applications. Computational modeling offers critical insights into intrinsic cardiac sources during the depolarization and repolarization phases of electrical activation. Mathematical representations of myocardial electrical activity, spanning multiple temporal and spatial scales, complement traditional experimental approaches, such as animal studies and clinical research. Moreover, computational models provide mechanistic insights into clinically relevant phenomena and offer a controlled setting to study how parameter changes quantitatively impact the entire system [3]. Patient-tailored computational models have significantly improved diagnostic, therapeutic, and prognostic outcomes in clinical practice [33].

Despite the significant potential of computational modeling, its clinical application is constrained by the high computational complexity and cost. Regarding the anatomical modeling of patient-specific hearts, most existing studies focus on individual atrial or ventricular structures, with limited research addressing the whole-organ electrophysiology of the four heart chambers. Additionally, myocardial electrophysiological modeling spans multiple scales, including the cellular, tissue/organ, and body surface levels.

Numerous studies have employed various methods to address forward problems in electrocardiography. Due to the irregular and complex geometries of the heart and torso, numerical techniques are frequently used. These methods can be broadly categorized into volume methods, which rely on differential equation techniques, and surface methods, which utilize integral function techniques [34]. The bidomain and monodomain models, grounded in Ohm’s law and Poisson’s equation, comprise a set of nonlinear partial differential equations. Numerical solutions for these models require discretization techniques to approximate the partial differential equations as linear systems [35]. Commonly used volume methods are the finite element method (FEM), finite difference method (FDM), and finite volume method (FVM). The FEM is well suited for the modeling of complex geometric structures, such as the heart and torso, due to its use of unstructured grids (e.g., triangles and tetrahedra), which accurately conform to intricate boundaries. In contrast, the FVM and FDM generally rely on structured grids (e.g., rectangles and cubes). These methods often require extensive mesh refinement to accommodate complex geometries, making it challenging to precisely capture anatomical boundaries and leading to reduced computational efficiency and increased numerical errors. The surface method refers to the boundary element method (BEM), which is a numerical technique used to compute the potential distribution on the torso surface based on the cardiac surface potentials. The BEM is particularly suited for problems involving homogeneous or layered media. Its main advantage lies in reducing the computational complexity by discretizing only the boundaries (e.g., the heart and torso surfaces) rather than the entire volume. This makes BEM an efficient approach to calculating body surface potentials, including the 12-lead ECG and body surface potential maps (BSPMs). However, the BEM requires the solution of boundary integral equations, which can be challenging in the presence of non-uniform media or complex geometries. Although the BEM reduces the dimensionality of the problem, it involves dense matrices, which can result in high computational costs for large-scale problems. Moreover, the BEM is not well suited for the direct simulation of electrophysiological processes within the myocardium, as it primarily focuses on boundary potentials.

The FEM was used to simulate electrical activity within the heart. Specifically, the cardiac domain was discretized into finite elements, and the bidomain equations were solved to obtain the spatiotemporal distribution of the transmembrane potential ($V_{m}$). The resulting heart surface potential distribution from the FEM solution was used as a boundary condition for the torso domain. The BEM was then employed to solve the Laplace equation in the torso domain and compute the body surface potentials. In COMSOL Multiphysics 6.2, separate physics interfaces were defined for the cardiac domain (FEM) and the torso domain (BEM). Coupling between the FEM and BEM was achieved by mapping the heart surface potentials from the FEM solution to the BEM boundary conditions. Unlike previous studies, we discovered that several different cardiac domains’ model parameters were applied in electrophysiological modeling. We calculated these parameters separately to achieve results that were more physiologically aligned with clinical observations. A set of parameters was chosen by comparing and analyzing the 12-lead ECG morphology. The position of the heart within the torso can influence the simulation outcomes of 12-lead ECG. To investigate this, the angle of the heart relative to the torso was adjusted to more accurately define its orientation in electrophysiological modeling. Furthermore, we expanded the previous electrophysiological modeling, which was based on a simplified heart–torso geometry, by incorporating a realistic heart–torso model [14,15,16]. Since the left atrium, right atrium, and ventricles in our heart model are isolated and unconnected, we added external stimulation to the mathematical model to establish a pathway for electrical signal propagation between the atria and ventricles.

The current study focused on normal physiological conditions to establish a foundation for future studies on pathological states. However, the model can be extended to simulate specific pathological conditions. For example, reduced blood flow in specific regions of the heart can be simulated by locally decreasing the tissue conductivity, modifying the action potentials and their durations, and reducing the conduction velocity. Setting the conductivity to a very low value effectively simulates non-excitable scar tissue regions. A conduction block can be simulated by modifying the diffusion coefficient in specific regions to model impaired electrical propagation.

A limitation of this study lies in representing T-wave heterogeneity in the 12-lead ECG when using a realistic heart–torso model. Typically, ventricular repolarization starts immediately after depolarization, producing a T-wave on the ECG. The process of ventricular repolarization differs from that of depolarization, occurring from the epicardium to the endocardium. In this study, both ventricular depolarization and repolarization extended from the endocardium to the epicardium, with the repolarization vector directed from the epicardium to the endocardium. Consequently, the direction of the T-wave on the ECG was the opposite to that of the main QRS-wave.

## 6. Conclusions

Existing electrophysiological models, which integrate bidomain equations with FHN models, use varying cardiac domain parameters. We evaluated multiple parameter sets and selected the one that matched a normal clinical ECG morphology. Additionally, we incorporated external current stimulation into the electrophysiological model. Existing electrophysiological models are limited to simplified whole-heart geometries. We extended this research by incorporating realistic heart–torso geometric models. To identify the heart’s position within the torso, we simulated how changes in the heart–torso angle influenced the 12-lead ECG.

The electrophysiological model developed in this study is easily adaptable to patient-specific cardiac domain parameters and capable of simulating ECG signals under pathological conditions. However, the accuracy of pathological simulations depends on the availability of detailed experimental data for parameter calibration. The current model excludes volume conduction effects from the bones, liver, lungs, and blood chambers. Future work will focus on extending the current model by incorporating patient-specific heart-torso models along with corresponding clinical and pathological data. In addition, the model focuses solely on electrical excitation and the resulting transmembrane ion currents, indicating that future studies should consider incorporating stretch-induced currents.

## Figures and Tables

**Table 1:** Different model parameters of the heart subdomains.

Parameter	SAN	Atria	AVN	His	BNL	PKJ	Ventricles	Reference
a	−0.60	0.13	0.13	0.13	0.13	0.13	0.13	Model 1 [14]
b	−0.30	0	0	0	0	0	0	
$c_{1}$	1000	2.6	2.6	2.6	2.6	2.6	2.6	
$c_{2}$	1.0	1.0	1.0	1.0	1.0	1.0	1.0	
d	0	1	1	1	1	1	1	
e	0.0660	0.0132	0.0132	0.0050	0.0022	0.0047	0.0060	
A (m V)	33	140	140	140	140	140	140	
B (m V)	−22	−85	−85	−85	−85	−85	−85	
k (s^−1^)	1000	1000	1000	1000	1000	1000	1000	
$\sigma_{e}$ (m S·m^−1^)	0.5	8	0.5	10	15	35	8	
$\sigma_{i}$ (m S·m^−1^)	0.5	8	0.5	10	15	35	8	
a	−1	0.13	0.13	0.13	0.13	0.13	0.13	Model 2 [15]
b	−0.29 × 10^−3^	0	0	0	0	0	0	
$c_{1}$	1.9	2.6	2.6	2.6	2.6	2.6	2.6	
$c_{2}$	1 × 10^−3^	1.0	1.0	1.0	1.0	1.0	1.0	
d	0	1	1	1	1	1	1	
e	0.060	0.010	0.010	0.0045	0.0028	0.0043	0.0050	
A (m V)	35	140	140	140	140	140	140	
B (m V)	−30	−85	−85	−85	−85	−85	−85	
k (s^−1^)	1000	1000	1000	1000	1000	1000	1000	
$\sigma_{e}$ (m S·m^−1^)	0.5	8	0.5	10	15	35	8	
$\sigma_{i}$ (m S·m^−1^)	0.5	8	0.5	10	15	35	8	
a	−1	0.13	0.13	0.13	0.13	0.13	0.13	Model 3 [16]
b	−0.29 × 10^−3^	0	0	0	0	0	0	
$c_{1}$	1.9	2.6	2.6	2.6	2.6	2.6	2.6	
$c_{2}$	1 × 10^−3^	1.0	1.0	1.0	1.0	1.0	1.0	
d	0	1	1	1	1	1	1	
e	0.035	0.020	0.010	0.0045	0.0028	0.0043	0.0050	
A (m V)	35	140	140	140	140	140	140	
B (m V)	−30	−85	−85	−85	−85	−85	−85	
k (s^−1^)	1000	1000	1000	1000	1000	1000	1000	
$\sigma_{e}$ (m S·m^−1^)	0.5	4	0.5	10	15	35	4	
$\sigma_{i}$ (m S·m^−1^)	0.5	4	0.5	10	15	35	4	

**Table 2:** Results of mesh convergence analysis tests.

Test Number	1	2	3	4
Heart domain mesh setting	Fine	Finer	Finer	Fine
Torso domain mesh setting	Normal	Normal	Fine	Fine
Left ventricular epicardial transmembrane potential (V)	0.053	0.0531	0.053	0.053
Relative error (%)	-	0.2	0.2	0
Left ventricular endocardial transmembrane potential (V)	0.0526	0.0526	0.0526	0.0526
Relative error (%)	-	0	0	0
P-wave amplitude (10^−5^ V)	0.0713	0.0716	0.0903	0.0903
Relative error (%)	-	0.4	20.71	21.04
QRS-wave amplitude (10^−5^ V)	3.1229	3.1104	3.7194	3.7194
Relative error (%)	-	0.4	16.37	16.04
T-wave amplitude (10^−5^ V)	−1.3451	−1.3462	−1.5295	−1.5295
Relative error (%)	-	0.08	11.98	12.06

## Data Availability

No new data were generated or analyzed in this study. The mathematical models and computational methods are described in sufficient detail within the article to allow replication.

## Abbreviations

The following abbreviations are used in this manuscript:

ECG	Electrocardiogram
FHN	Fitzhugh–Nagumo
BSPMs	Body surface potential maps
SAN	Sinoatrial node
AVN	Atrioventricular node
His	His bundle
BNL	Bundle branches
PKJ	Purkinje network
CBIC	Centre for Integrative Biomedical Computing
BB	Bachmann’s Bundle
FEM	Finite element method
FDM	Finite difference method
FVM	Finite volume method
BEM	Boundary element method
